# Impact of a Regulation Restricting Critical Antimicrobial Usage on Prevalence of Antimicrobial Resistance in *Escherichia coli* Isolates From Fecal and Manure Pit Samples on Dairy Farms in Québec, Canada

**DOI:** 10.3389/fvets.2022.838498

**Published:** 2022-02-17

**Authors:** Maud de Lagarde, John M. Fairbrother, Marie Archambault, Simon Dufour, David Francoz, Jonathan Massé, Hélène Lardé, Cécile Aenishaenslin, Marie-Ève Paradis, Jean-Philippe Roy

**Affiliations:** ^1^Department of Clinical Sciences, Faculty of Veterinary Medicine, Université de Montréal, Saint-Hyacinthe, QC, Canada; ^2^Regroupement FRQNT Op+lait, Saint-Hyacinthe, QC, Canada; ^3^OIE Reference Laboratory for Escherichia coli, Faculty of Veterinary Medicine, Université de Montréal, Saint-Hyacinthe, QC, Canada; ^4^Swine and Poultry Infectious Research Center (CRIPA-FQRNT), Faculty of Veterinary Medicine, Université de Montréal, Saint-Hyacinthe, QC, Canada; ^5^Department of Pathology and Microbiology, Faculty of Veterinary Medicine, Université de Montréal, Saint-Hyacinthe, QC, Canada; ^6^Department of Clinical Sciences, Ross University School of Veterinary Medicine, Basseterre, Federation of Saint Christopher and Nevis; ^7^Association des Médecins Vétérinaires Praticiens du Québec, Saint-Hyacinthe, QC, Canada

**Keywords:** ESBL/AmpC, cattle, calf/calves, bacterial clone, *Escherichia coli*, antimicrobial resistance

## Abstract

To tackle antimicrobial resistance (AMR), one of the major health threats of this century, the World Health Organization (WHO) endorsed a global action plan in 2015. This plan calls countries to develop national actions to address AMR. The province of Québec, Canada, adopted a new regulation on the 25^th^ of February 2019, to limit the use in food animals of antimicrobials of very high importance in human medicine. We aimed to establish the impact of this regulation by comparing the AMR situation in dairy cattle in Québec ~2 years before and 2 years after its introduction. We sampled calves, cows, and the manure pit in 87 farms. Generic and putative ESBL/AmpC *E. coli* were tested for susceptibility to 20 antimicrobials. Logistic regression was used to investigate whether the probability of antimicrobial resistance differed between isolates obtained from the pre and post regulation periods by sample type (calves, cows, manure pit) and in general. To identify AMR genes dissemination mechanisms, we sequenced the whole genome of 15 generic isolates. In the generic collection, at the herd level, the proportion of multidrug resistant (MDR) isolates, decreased significantly from 83 to 71% (*p* = 0.05). Folate inhibitor and aminoglycoside resistances demonstrated a significant decrease. However, when analyzed by sample type (calves, cows, manure pit), we did not observe a significant AMR decrease in any of these categories. In the ESBL/AmpC collection, we did not detect any significant difference between the two periods. Also, the general resistance gene profile was similar pre and post regulation. We identified both clonal and plasmidic dissemination of resistance genes. In conclusion, as early as 2 years post regulation implementation, we observed a significant decrease in MDR in the dairy industry in Quebec in the generic *E. coli* collection with folate inhibitor and aminoglycoside resistances showing the most significant decrease. No other significant decreases were yet observed.

## Introduction

Building sustainable food systems relies on effective antimicrobials being available to treat infections and ensure animal welfare. However, it is now well-recognized that antimicrobial resistance (AMR) threatens environmental, animal and public health and there is no more time to waste (1). Indeed, in 2015, recognizing the urgent need to tackle AMR, the membership of Food and Agriculture Organization (FAO), World Organization for Animal Health (OIE) and World Health Organization (WHO) endorsed a global action plan on AMR (GAP) (2). In 2016, the United Nations (UN) General Assembly reaffirmed the GAP as the guideline for fighting AMR and committed themselves to supporting and implementing it at the global, national, and regional levels (3). The GAP recognize in its fourth objective that one of the main actions for the different health actors, to contain AMR, is the judicious use of antimicrobials to reduce selective pressure on microorganisms. Indeed, all over the world, there is substantial misuse and/or overuse of antimicrobials in humans and food animals (4). WHO's GAP acknowledged laws and regulation as essential tools for ensuring the application of national standards to optimize the use of antimicrobials in human and animal health. This includes a call for all countries to develop and implement collaborative, multisectoral national action plans to address AMR in each country.

In this context, the province of Québec (Canada) adopted a new regulation on the 25^th^ of February 2019, to restrict usage of category 1 antimicrobials of the Health Canada classification (5) in production animals (6). Briefly, Health Canada classified antimicrobials as function of their importance for human health. Category 1 antimicrobials are those of very high importance for humans based on two selective criteria: they are identified as the preferred option of treatment of serious human infections and there is no other (or limited) available alternative. The new regulation prohibits the use of these antimicrobials for preventive purposes in food-producing animals and restricts their usage for curative purposes unless it has been justified (e.g., with an antimicrobial susceptibility test) that there are no other effective alternative drugs available of lower importance (7). This regulation intends to limit the use of category 1 antimicrobials to rare curative cases. The goal is to limit selective pressure by antimicrobials, which should lead to an eventual decrease in AMR. However, implementation of rules without monitoring their effect in the field could result in applying restrictive and ineffective pressure on the food industry, which is always submitted to fierce competition.

In 2017, prior to the regulation implementation, the portrait of both antimicrobial use (AMU) and AMR in 101 dairy farms in Québec has been documented. It was demonstrated that the category 1 antimicrobials used on dairy farms were mainly third generation cephalosporins, fluoroquinolones and polymyxins. The intramammary route was the most frequently observed. The median herd was using 88 defined course doses (DCDbovCA) /100 cows-years of these latter antimicrobials (8). A change of AMU is expected after the regulation implementation and might influence AMR. Extended spectrum β-lactamase/AmpC (ESBL/AmpC) producing *E. coli* were found in either fecal or manure pit samples of 85% of these farms (9). As these results were published by our research team, we had an excellent comparison point to establish the impact of the regulation on the AMR in the dairy industry in Québec. Moreover, our team recently demonstrated an average category 1 AMU herd-level reduction of 80% of prescription following the implementation of the new regulation (10).

The objectives of the current paper were therefore: (i) to report the AMR situation ~2 years after the regulation implementation; and (ii) to compare these results with the assessment that was performed 2 years prior to the regulation (9). The overall outcome was to determine if this regulation was beneficial for the dairy industry and to provide scientific evidence for others wishing to use a similar approach.

## Materials and Methods

### Selection of Herds and Sample Collection

**Figure 1:**
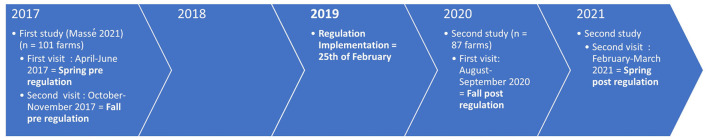
Timeline of the sampling compared to the regulation implementation. Spring pre regulation and fall pre regulation were part of a previous study [Massé et al. (9)] that aimed at establishing the portrait of AMR in dairy farms in Québec, Canada. Spring post regulation and fall post regulation are the period of sampling ~2 years after the implementation of the regulation in 87 of the same herds and used for comparison.

We used an observational descriptive cohort study on commercial dairy farms. Prior to initiating the research, the research protocol was approved by the Animal Use Ethics and the Research Ethics Committees of the Université de Montréal (20-Rech-2085). Written informed consent was obtained from the owners for the participation of their animals in this study. To enable a proper comparison between the period pre and post regulation implementation, we sampled the same herds as those sampled in 2017 to establish AMR prevalence in dairy farms in Québec (8, 9). The previous 101 farms, located in the three main dairy areas of Québec, Canada (Montérégie, Center-du Québec and Estrie), were contacted by a member of the previous research team (HL) in July 2020 and asked to participate to a second set of sampling. Following recruitment, two sampling visits were made, firstly between August and September 2020 and secondly between February and March 2021. Figure 1 clarifies the timeline of the four periods of sampling and the time of the regulation implementation.

The sampling protocol was followed as previously described (9). Briefly, on each visit, fecal samples were collected from five pre-weaned calves and mixed to obtain a composite sample. Then fecal samples of five lactating cows were also collected and mixed to obtain another composite sample. On each farm, a convenience sample was assembled based on accessibility of the calves and cows. Fecal samples were obtained directly from the rectum for calves and freshly voided cow feces were obtained from the floor. A composite manure sample was also collected from two convenient locations in the manure pit. For each of these six composite samples, approximately 25 g of feces or manure were placed in a 50 mL sterile tube and stored immediately on ice at the farm. Samples were processed in the laboratory within <24 h. A preservative medium (peptone water with 30% glycerol) was added to the sample at a 1:1 volume-to-weight ratio; samples were then homogenized and frozen at −80°C.

### Bacterial Isolation and *Escherichia coli* Identification

#### Generic Collection

To accurately compare the period pre and post regulation implementation, we used the same protocol for bacterial isolation as the one we used in the initial AMR prevalence study (9). Briefly, 1 g of each composite sample was mixed in phosphate buffer saline and then streaked on MacConkey plates and incubated overnight at 37°C. One lactose positive colony was chosen for each composite sample was subcultured on Columbia agar with 5% sheep blood (Oxoid, Canada), and incubated overnight at 37°C. The identification of isolates as *E. coli* was confirmed by MALDI-TOF MS using a Microflex LT instrument (Bruker Daltonics, Germany).

#### ESBL/AmpC Collection

To allow an accurate comparison between the period pre and post regulation implementation, we also used the same protocol for bacterial isolation from our initial AMR prevalence study (9). Briefly, composite fecal samples were processed according to the laboratory protocol of the European Union Reference Laboratory on Antimicrobial Resistance which allowed the recovery of ESBL-, AmpC- and carbapenemase-producing *E. coli* from composite fecal samples. The protocol is available online at https://www.eurl-ar.eu/protocols.aspx. Briefly, 1 g of each composite fecal or manure sample was added to 9 mL of Buffered Peptone Water, then incubated at 37°C for 20 h. One loop (10 μl) was streaked onto a MacConkey agar plate containing 1 mg mL^−1^ of cefotaxime, then incubated at 44°C for 20 h. Lactose positive colonies were subcultured on Columbia agar with 5% sheep blood, and then incubated overnight at 37°C. Identification of *E. coli* was confirmed by MALDI-TOF MS. Composite samples with at least one *E. coli* colony isolated with this technique were labeled as presumptive ESBL/AmpC *E. coli*.

All *E. coli* selected in both collections were incubated for 24 h at 37°C in Luria-Bertani (LB) broth then mixed 50:50 with 30% glycerol and stored at −80°C.

### Antimicrobial Susceptibility Testing

The minimum inhibitory concentrations (MIC) against 20 antimicrobials representing 12 classes of antimicrobials were determined on all isolates belonging to both collections. The broth microdilution method was used with the commercially available panels (Sensititre CMV4AGNF and BOPO7F) (Thermo Fisher scientific, Canada) following manufacturer recommendations in accordance with the Clinical Laboratory Standards Institute standards (11). For each antimicrobial, dilution range, class, breakpoint, MIC and category classification are available in Table 1. Isolates were defined as susceptible, intermediate, or resistant with the same criteria as previously described (9). Briefly, we used the CLSI M100 (12) (*Enterobacteriaceae*: amoxicillin/clavulanate, azithromycin, ampicillin, cefoxitin, ceftriaxone, chloramphenicol, ciprofloxacin, gentamicin, meropenem, nalidixic acid, sulfisoxazole, tetracycline and trimethoprim/sulfamethoxazole), CLSI VET08 (13) (ceftiofur, danofloxacin, enrofloxacin, and spectinomycin), or CIPARS (14) (streptomycin) clinical breakpoints. A MIC breakpoint was not available for neomycin, thus the epidemiological cut-off value from European Committee on Antimicrobial Susceptibility Testing (EUCAST) was used (MIC ≥16 μg mL^−1^ was defined as resistant). There were no valid florfenicol clinical breakpoints for *Enterobacteriaceae* and the tested concentrations (0.25–4 μg mL^−1^) did not include the European epidemiological cut-off of 16 μg mL^−1^, therefore no interpretation was attempted. For subsequent analyses, intermediate and resistant isolates were grouped together and labeled as resistant. *Enterococcus faecalis* ATCC 29212, *Escherichia coli* ATCC25922, *Staphylococcus aureus* ATCC 29213 and *Pseudomonas aeruginosa* ATCC 27853 were used as reference strains for batch controls. *Escherichia coli* ATCC 25922 was used as a daily control.

**Table 1:**
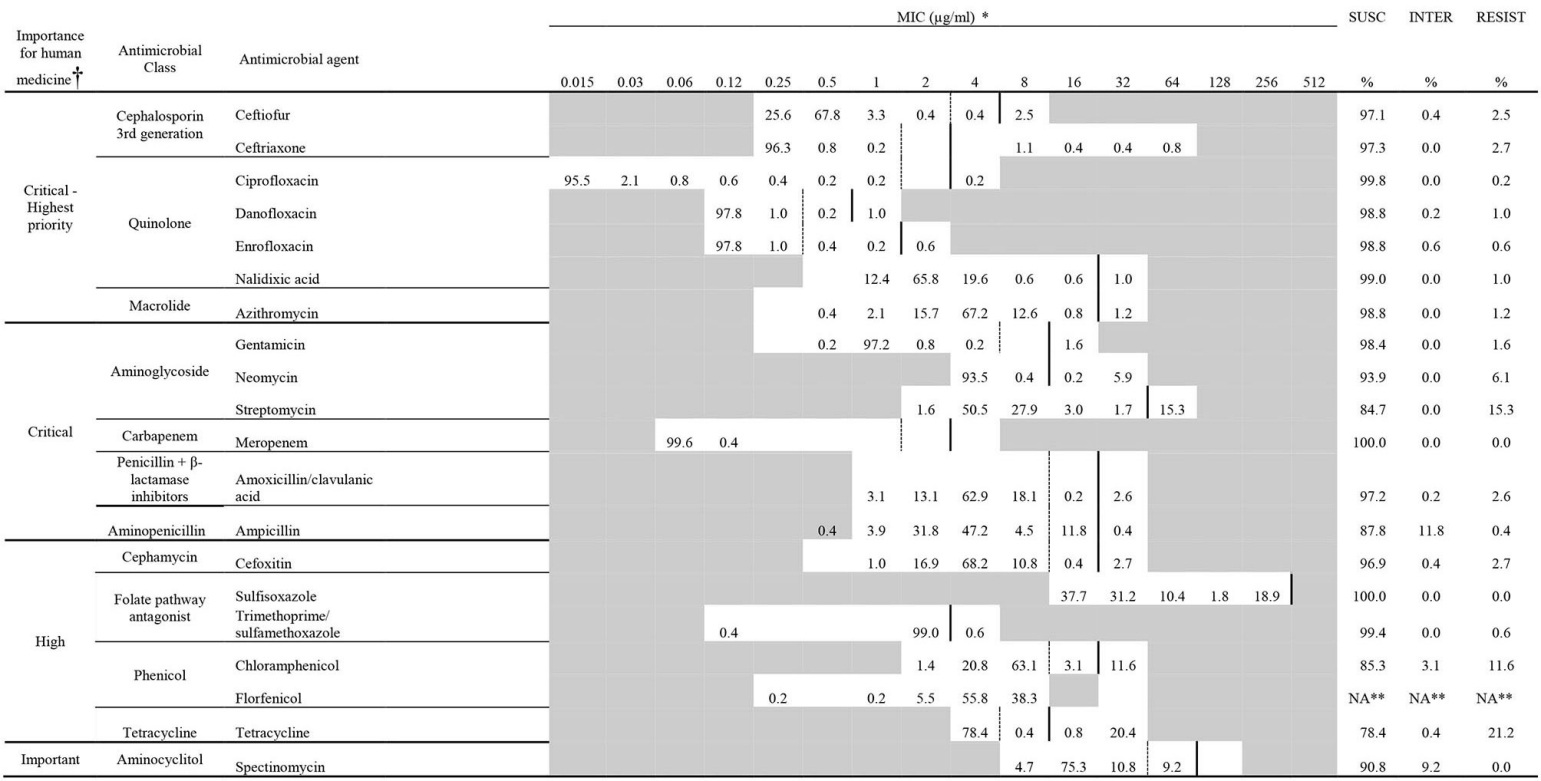
Minimum inhibitory concentration for medically important antimicrobials, according to the WHO, of 509 *Escherichia coli* isolated in the generic collection from calf or cow feces or manure pit of 87 dairy farms in Québec, Canada.

*Numbers indicate percentages of isolates. White areas are concentrations of antimicrobials tested by the broth microdilution method. Dashed and plain lines represent threshold used to define intermediate and resistant clinical breakpoints, respectively. †Importance of antimicrobials according to World Health Organization. **Florfenicol has no valid clinical breakpoints for Enterobacteriaceae and the concentration of 0.25 to 4μg mL−1 did not include the European epidemiological breakpoint of 16 μg mL−1, thus no interpretation could be given.

For statistical analyses, intermediate and resistant isolates were combined and designated as resistant. Multidrug resistance (MDR) was defined as acquired resistance to at least one agent in three or more antimicrobial classes and extensively drug resistance was defined as resistant to at least 1 agent in all but 2 or fewer antimicrobial classes (15) as previously defined (9).

In the ESBL/AmpC collection we determined the ESBL/AmpC phenotype based on the results of the MIC. An isolate was called “ESBL” if it was resistant to ceftriaxone or ceftiofur, susceptible to meropenem, susceptible to cefoxitin and susceptible to amoxicillin/clavulanic acid. An isolate was called “AmpC” if it was resistant to ceftriaxone or ceftiofur, susceptible to meropenem, resistant to cefoxitin and resistant to amoxicillin/clavulanic acid. An isolate was called “ESBL/AmpC” if it was resistant to ceftriaxone or ceftiofur, susceptible to meropenem, resistant to cefoxitin and susceptible to amoxicillin/clavulanic acid. An isolate was called “other phenotype” if it was resistant to ceftriaxone or ceftiofur, susceptible to meropenem, susceptible to cefoxitin and resistant to amoxicillin/clavulanic acid or if it was susceptible to ceftriaxone and ceftiofur, susceptible to meropenem, resistant or susceptible to cefoxitin and resistant to amoxicillin/clavulanic acid.

### Antimicrobial Genotyping

Whole genome sequencing (WGS) was used on a subset of isolates of the generic collection to determine the genetic basis of the observed AMR. Due to financial and logistic restrictions, we sequenced 15 isolates in total. The selection was based on relevant phenotypes with the following criteria: isolates resistant to 8 or more antimicrobials classes (aminoglycosides and aminocyclitols were considered two different classes for this selection) (*n* = 4), isolates identified as harboring an ESBL (*n* = 4) or an AmpC (*n* = 6) phenotype, and an isolate resistant to danofloxacin and enrofloxacin. Briefly, genomic DNA was extracted using QIAamp DNA Mini Kit for DNA following manufacturer's guidelines (Qiagen, Hilden, Germany). We performed WGS on the Illumina (San Diego, CA) iSeq100 platform with 2 × 150 paired end runs after library preparation with the Illumina DNA prep kit (former Nextera Flex kit), according to the manufacturer's instructions. Illumina platform was used to assemble genomes using SPADES 3.9.0. An assembly was rejected if the number of contigs (>500 pb) was >400 or if the N50 was <50,000. Details of data assembly quality are available in Supplementary Table S1. To search AMR genes and point mutations, Res Finder 4.0 (16) and Point Finder (17) bioinformatics tools from the Center of Genomic Epidemiology (CGE) platform (http://www.genomicepidemiology.org/) were used. To complete the analysis, we also used the CARDS database (18). Multi locus sequence typing (MLST) (19), O and H serotype (20) and core genome MLST (cgMLST) (21) were determined by the analysis of generated FASTA files using the Center of Genomic Epidemiology (CGE) platform (http://www.genomicepidemiology.org/ accessed on 11/15/2021). Phylogroups were determined with in-silico PCR using the Clermont Typing platform (http://clermontyping.iame-research.center/ accessed on 11/15/2021) (22).

### Statistical Analysis

For all statistical analyses, the unit of analysis was the composite sample obtained from different origins (calves, cows, or manure pit), time periods (pre and post regulation), seasons (fall 1 and 2 or spring 1 and 2 visits), and herds. Each sample was represented by one *E. coli* isolate. We also conducted herd-period level analyses. For these latter analyses, if one of the 6 isolates obtained in each herd (pre or post regulation) was found positive for an outcome, the herd was considered positive for this outcome during that period.

#### Effect of Regulation Implementation on Antimicrobial Resistance

In the generic collection, we investigated whether the probability of resistance to a given antimicrobial or the probability for an isolate of being MDR differed between isolates obtained from the periods pre and post regulation. Because a season effect was detected in the previous study (9), we also compared the pre and post regulation periods by season (spring pre *vs*. spring post regulation and fall pre *vs*. fall post regulation). In the ESBL/AmpC collection, we investigated whether the probability of a sample to harbor a putative ESBL/AmpC *E. coli* differed between isolates obtained from the same periods. As calves demonstrated the greatest risk to present AMR in the previous study (9), we calculated the probabilities for each outcome by sample type (calves, cows, or the manure pit) and at the herd level. For all these analyses, we used a logistic regression model with susceptibility *vs*. resistance to a given antimicrobial or the MDR status or the growth of an *E. coli* on the cefotaxime plate as outcome variable. Sample type and periods (pre *vs*. post regulation) were used as fixed predictors and the model was considered to be a generalized mixed model in which a herd random effect was included to account for clustering of samples or isolates within herds (SAS, PROC GLIMMIX. Cary, NC, US). Tukey-Kramer adjustment was used to adjust for multiple comparisons. An alpha of 0.05 was chosen to define statistically significant results.

#### Effect of Regulation Implementation on Numbers of Antimicrobials to Which an Isolate Was Resistant to and on the ESBL Profile of Isolates From the ESBL/AmpC Collection

A generalized linear mixed model (SAS, PROC GLIMMIX, Cary, NC, US) was used to investigate whether the regulation implementation could influence the number of antimicrobials to which an isolate was resistant to and the ESBL profile of isolates from the ESBL/AmpC collection. In this model, a negative binomial distribution with a log link was used. The outcome was the number of antimicrobial classes to which an isolate was defined as resistant (0 to 10) or the ESBL profile (1–4). The predictor was either the origin of the samples (calves, cows, or manure pits) or the period (pre vs. post regulation implementation) and a random herd intercept was included to account for clustering of isolates by herd. A Tukey-Kramer test was applied to adjust for multiple comparisons and an alpha of 0.05 was used.

## Results

### Selection of Herds and Sample Collection

Eighty-seven of the 101 farms accepted to participate in the post regulation set of sampling. Descriptive data for all samplings (pre and post regulation) on the 87 farms are available in Table 2.

**Table 2:** Descriptive statistics of cattle sampled in 87 farms dairy farms in Québec, Canada, per period of sampling (pre and post regulation).

	**Calf**					**Cow**				
	**Number**	**Mean age**	**Median age**	**Youngest (in days)**	**Oldest (in days)**	**Number**	**Mean lactation**	**Median lactation**	**Min lactation**	**Max** **lactation**
Fall pre regulation	339	27	21	1	150	435	2.6	2	1	10
Spring pre regulation	274	29	25	1	100	434	2.4	2	1	9
Fall post regulation	350	31	28	1	170	435	2.6	2	1	8
Spring post regulation	337	28	24	1	120	435	2.7	2	1	9

For the sampling post regulation, manure pits were emptied approximately 4 months before the first visit. During the second visit, all manure pits were frozen, therefore we sampled the end of the drainpipe or the gutter. In this sampling, among the 516 fecal composite samples obtained, we recovered 509 *E. coli* isolates, in the generic collection. Indeed, 7 samples from manure pits, sampled in fall 2020, did not yield any lactose positive colonies. We also recovered 162 putative ESBL/AmpC *E. coli* in this putative ESBL/AmpC collection.

### AMR Situation Approximately 2 Years After the Regulation Implementation

#### Generic Collection

**Figure 2:**
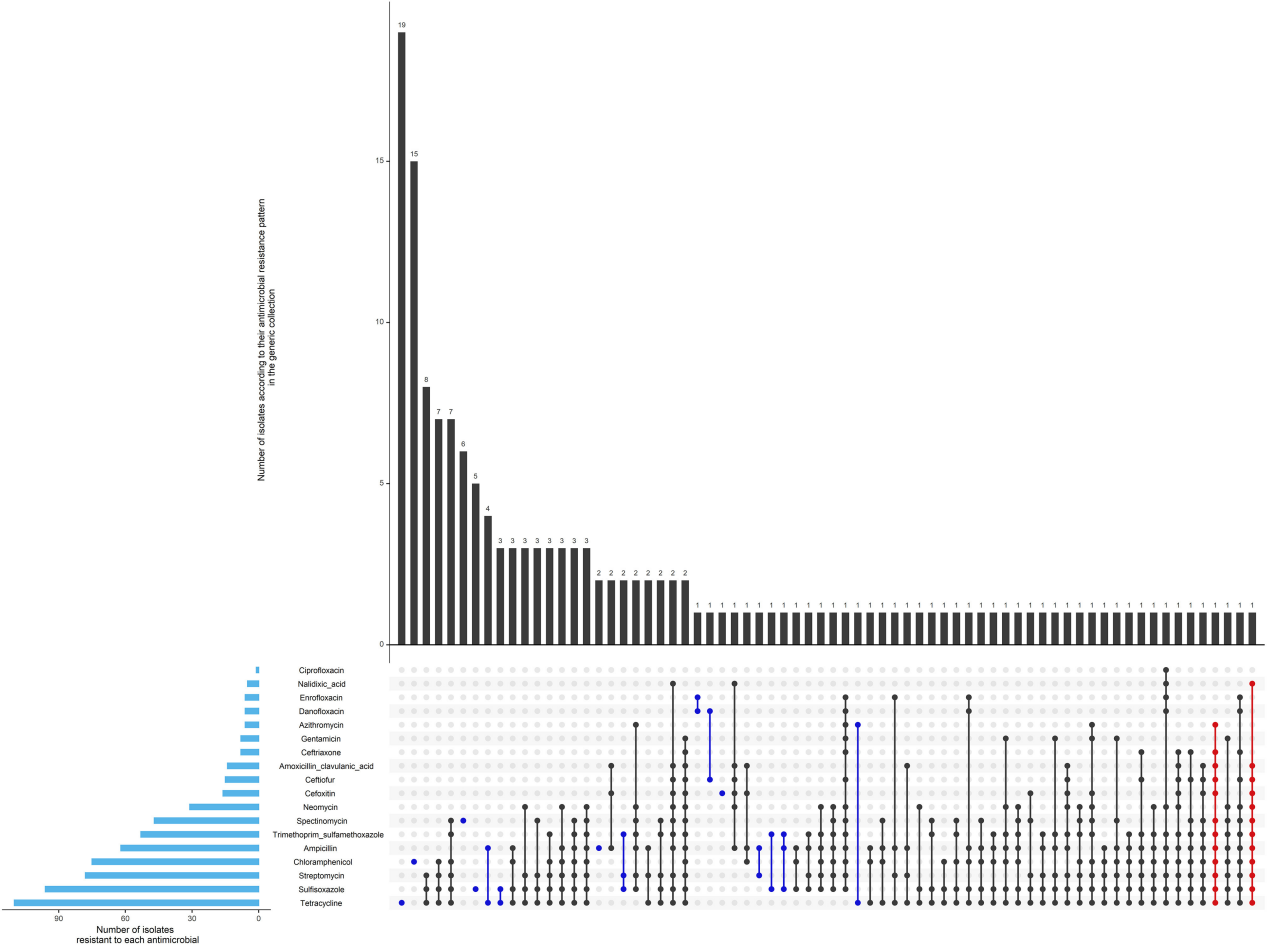
Antimicrobial resistance pattern of 509 isolates of the generic collection from calf, cow feces or manure pit of 87 dairy farms in Québec, Canada in 2020–2021. The horizontal blue bars represent the frequency of isolates resistant to each antimicrobial. An antimicrobial pattern is represented by the linked dots. Black dots represent the MDR patterns, and red dots represent the XDR patterns. The vertical bars represent the frequency of isolates for each antimicrobial pattern. 352 (69%) isolates were susceptible to all antimicrobials (not represented in the figure).

Most isolates (69%) were pan susceptible as presented in Table 1 and Figure 2. No resistance to meropenem (carbapenem class) was detected, although resistance to each of the other antimicrobials were observed at least once. Two isolates were considered extensively resistant (Figure 2). They were both identified in calves, however, not during the same period and not in the same farm.

**Figure 3:**
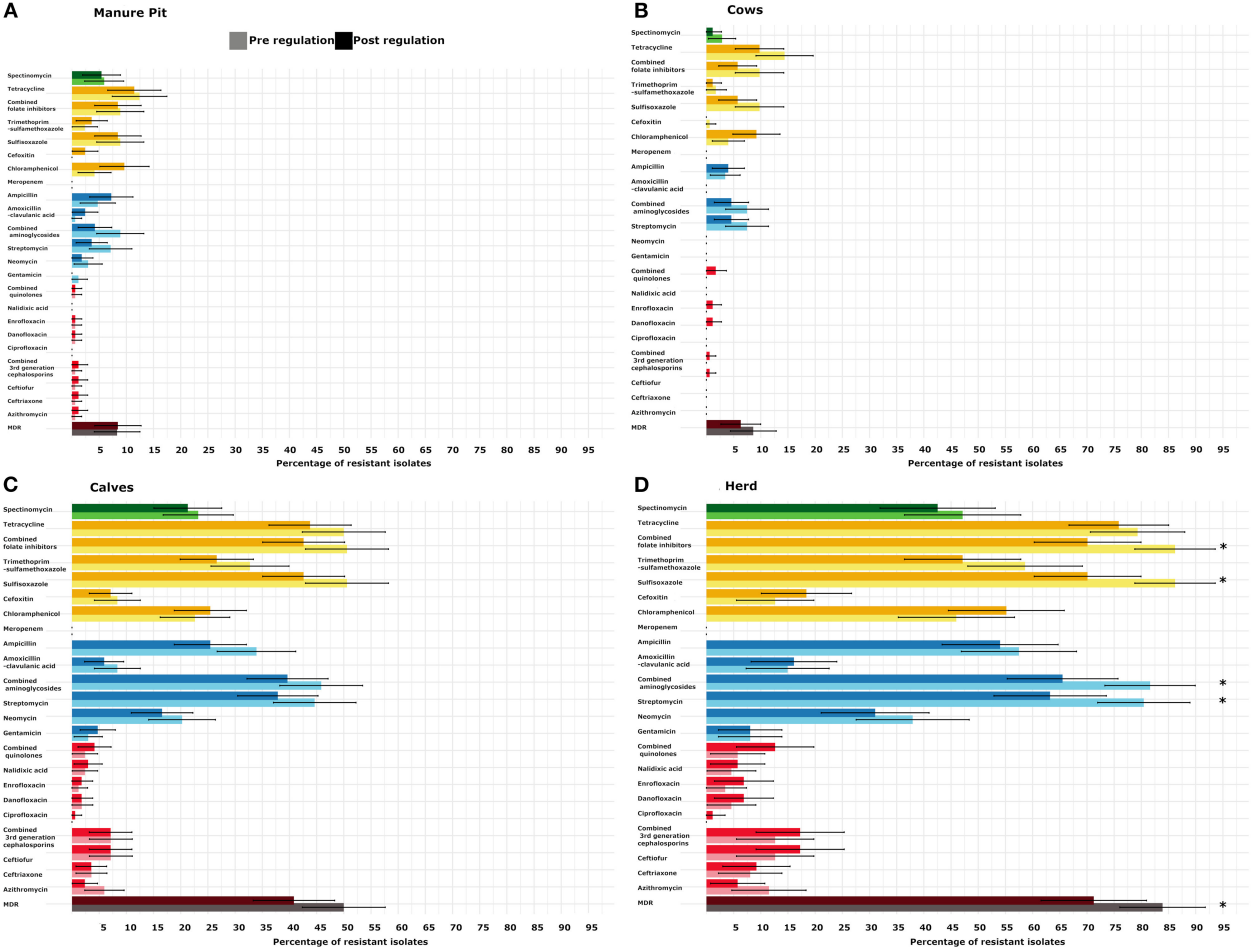
Comparison of the proportion of isolates with at least one resistant (intermediate and resistant combined) *Escherichia coli* per period [pre- (light) and post-regulation (dark)] and sample type [manure pit **(A)**, cows **(B)**, calves **(C)**] or for the whole herd **(D)** from 87 dairy farms from Québec, Canada. On each farm, between 4 and 6 *E. coli* were tested for each antimicrobial. In dark and light burgundy, MDR: multidrug resistant (resistant to 3 classes of antimicrobial or more). In dark and light red, critical high priority antimicrobials for human medicine. In dark and light blue, critical priority antimicrobials for human medicine. In dark and light yellow, high priority antimicrobials for human medicine. In dark and light green: antimicrobials important for human medicine, SPE: spectinomycin. The importance of antimicrobial for human medicine was defined according to World Health Organization.

**Figure 4:**
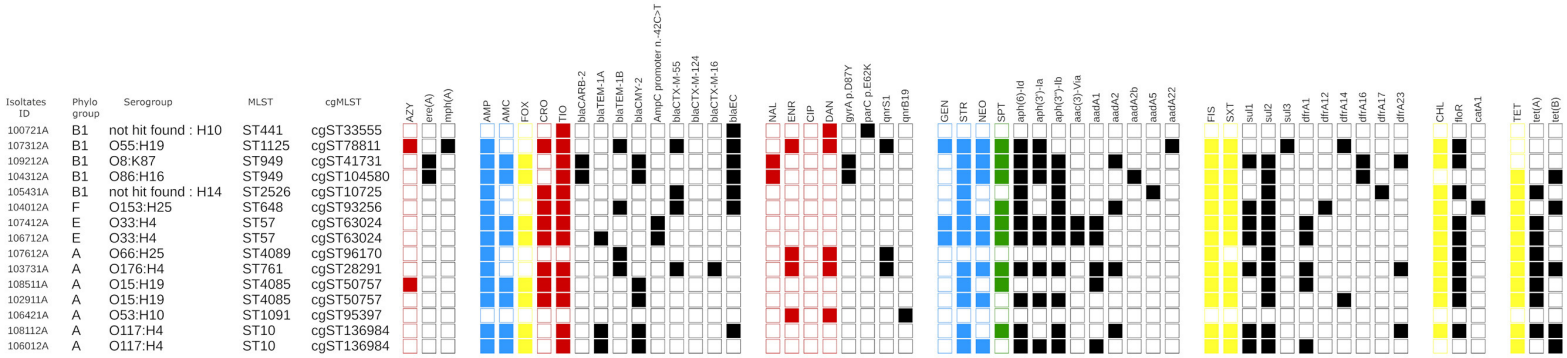
Phylogenic, phenotypic, and genotypic characteristic of 15 isolates from the generic collection from 87 dairy farms from Québec, Canada in 2020–2021 determined by whole genome sequencing. In red, critical high priority antimicrobials for human medicine, AZM, azithromycin; CRO, ceftriaxone; TIO, ceftiofur; CIP, ciprofloxacin; DAN, danofloxacin; ENR, enrofloxacin; NAL, nalidixic acid; in blue, critical priority antimicrobials for human medicine; GEN, gentamicin; NEO, neomycin; STR, streptomycin; AMC, amoxicillin-clavulanic acid; AMP, ampicillin; MEM, meropenem. In yellow, high priority antimicrobials for human medicine, CHL, chloramphenicol; FOX, cefoxitin; FIS, sulfisoxazole; SXT, trimethoprim-sulfamethoxazole, FOL, combined folate inhibitors; TET, tetracycline; in green, antimicrobials important for human medicine, SPT, spectinomycin. The importance of antimicrobial for human medicine was defined according to World Health Organization. The presence of a characteristic is noted by a full square. In the *bla*_*EC*_ family we recovered *bla*_*EC*−13_ (*n* = 2), _14_ (*n* = 1), _15_ (*n* = 2), _18_ (*n* = 1) and _19_ (*n* = 1).

As shown in Figure 3D, the highest levels of resistance, in herds, were toward tetracycline (76%), sulfisoxazole (70%), and streptomycin (63%). The most common AMR patterns were tetracycline (3.7%) and chloramphenicol (2.9%). The most prevalent MDR pattern was tetracycline-sulfisoxazole-streptomycin (1.6%). The most frequently observed resistance genes in tested isolates, identified with the WGS (*n* = 15), were responsible for tetracycline [*tet(A), tet(B)]*, sulfisozaxole (*sul1, sul2)*, and streptomycin *aph(6)-Id, aph(3')-Ia, aph(3”)-Ib, aadA1, aadA2*. The AmpC phenotype was associated mainly with *bla*_*CMY*−2_(*n* = 6/8). A mutation in the promoter of the AmpC gene was responsible for the AmpC phenotype in the remaining isolates (*n* = 2/8). The ESBL phenotype was associated with *bla*_*CTX*−*M*−55_ *(n* = *3/4)* and *bla*_*CTX*−*M*−124_ (*n* = 1/4). The *bla*_*EC*_ family was detected with CARDS in 7/15 isolates [*bla*_*EC*−13_ (*n* = 2), _14_ (*n* = 1), _15_ (*n* = 2), _18_ (*n* = 1) and _19_ (*n* = 1)]. All genes identified in the sequenced isolates are reported in Figure 4. There was a 100% correlation between phenotype resistance and associated resistance genes for all antimicrobials, except for azithromycin and danofloxacin with correlation of 80 and 93%, respectively. We also identified genes responsible for resistance to disinfectant (*qacE* or *sitABCD* or both) in 10/15 isolates that were sequenced.

As illustrated in Figure 4, sequenced isolates showed a diversity of phylogroups, MLST, serogroup and cgMLST. However, three pairs of isolates had the same phylogenetic characteristics (107412A and 106712A, 108511A and 102911A, 108112A and 106012A). Based on this analysis they could be considered as clones. They belong to different farms, and periods, although they were all recovered in calf samples. This suggests a possible clonal dissemination of the most resistant isolates in the calf's population. Their replicon profile, illustrated in Supplementary Figure S1, was nevertheless not identical, thus explaining their differences in resistance profile. On the other hand, the replicon that we identified among other isolates, were often similar, with an omnipresence of the replicon IncFIB. IncFIA and IncFII were present in, respectively, 7/15 and 13/15 isolates. Although, we could not circularize the plasmid sequenced and, therefore, we could not assign one gene to one plasmid, these data suggest that, in this *E. coli* population, AMR genes were also spread through horizontal transfer.

#### ESBL/AmpC Collection

**Figure 5:**
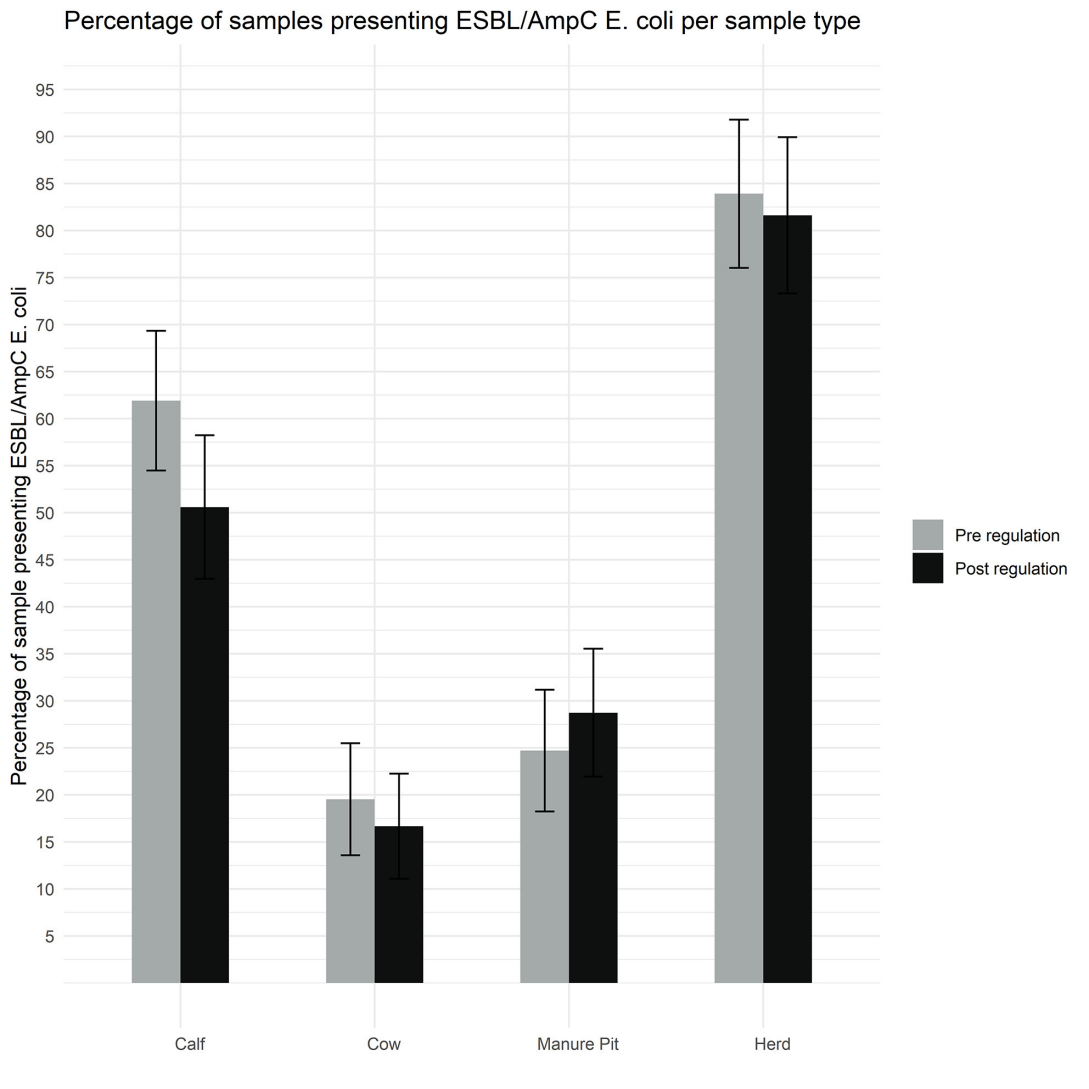
Comparison of the proportion of isolates with a putative ESBL/AmpC *Escherichia coli* per sample type (manure pit, cows, calves, herd) from 87 dairy farms from Québec, Canada, pre (gray) and post (black) regulation implementation.

**Figure 6:**
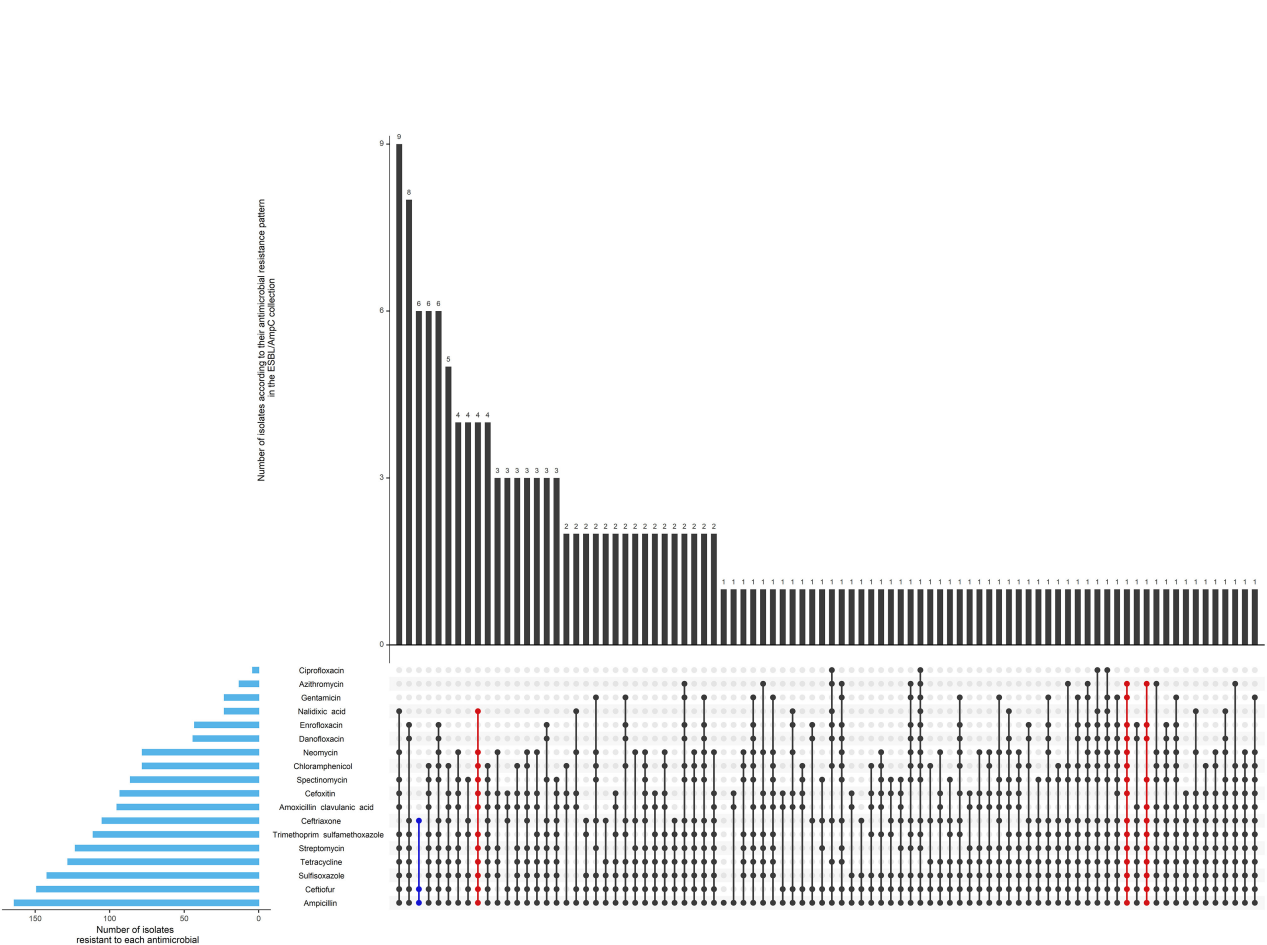
Antimicrobial resistance pattern of 162 isolates of the ESBL/AmpC collection from calf, cow feces or manure pit of 87 dairy farms in Québec, Canada in 2020–2021. The horizontal blue bars represent the frequency of isolates resistant to each antimicrobial. An antimicrobial pattern is represented by the linked dots. Black dots represent the MDR patterns, and red dots represent the XDR patterns. The vertical bars represent the frequency of isolates for each antimicrobial pattern.

As shown in Figure 5, 82% (71/87) of herds were positive for ESBL/AmpC-producing *E. coli* in at least one sample during the post regulation period. According to our definitions 39% (63/161) of isolates had an ESBL profile, 48% (77/162) had an AmpC profile, 2% had an ESBL/AmpC profile and 11% (17/161) isolates had a profile designated as “other.” Only 6 isolates were not MDR, and 6 isolates were extensively resistant (Figure 6).

### Impact of Regulation on the AMR Situation

For statistical comparison we excluded farms that were sampled only pre regulation, and therefore used 87 farms. In the generic collection, 511 and 509 *E. coli* isolates were available for the pre and post regulation period, respectively. In the ESBL/AmpC collection 181 and 162 *E. coli* isolates were available for the pre and post regulation period, respectively.

#### Generic Collection

As shown in Figures 3A–C, there were no statistical difference in the proportion of resistant isolates for antimicrobials tested between the pre and post regulation periods for the samples originating from manure pits, cows, or calves. However, at the herd level, the recovery percentage of MDR *E. coli* was statistically lower post regulation implementation (2.2 times lower odds; 95% CI (1.4–3.3); *p* = 0.05; Table 3). The recovery percentages of isolates positive for resistance to streptomycin and sulfisoxasole were also lower at the herd-level (odds ratio and *p*-value are available in Table 3).

**Table 3:** Parameter estimates and odds ratio from logistic regression models, for various outcomes, and using either the sample (feces from calves, feces from cows, manure pit sample) or herd as unit of analysis, based on the results of a cross-sectional study performed on 87 farms sampled in Québec, Canada between 2017 and 2021.

**Outcome**	**Period compared**	**Sample type**	**Odd ratio**	**95% CI**	**P-value**
MDR	Pre vs. post regulation	Herd	2.2	1.5–3.3	0.05
Streptomycin	Pre vs. post regulation	Herd	2.4	1.7–3.4	0.01
Sulfizoxasole	Pre vs. post regulation	Herd	2.9	1.9–4.6	0.01
MDR	Fall pre vs. fall post regulation	Calves	3.0	2.1–4.3	0.03
Sulfizoxasole	Fall pre vs. fall post regulation	Calves	2.8	2.0–3.9	0.04
MDR	Fall pre vs. fall post regulation	Herd	2.4	1.6–3.4	0.02
Streptomycin	Fall pre vs. fall post regulation	Herd	2.5	1.7–3.5	0.01
Sulfizoxasole	Fall pre vs. fall post regulation	Herd	2.1	1.6–3.0	0.02
Trimethoprim-sulfamethoxazole	Fall pre vs. fall post regulation	Herd	2.1	1.4–3.0	0.05

Comparisons between pre *vs*. post regulation spring samples and pre *vs*. post regulation fall samples were also performed (Supplementary Figures S2, S3). No significant difference was observed between spring samples. However, the recovery percentages of herds positive for the presence of an MDR isolate and the resistance to streptomycin and sulfisoxasole and trimethoprim-sulfisoxasole were lower in post compared to pre regulation fall samples (odds ratio and *p*-value are presented in Table 3). Moreover, the recovery percentage of MDR isolates in calf samples and the resistance to sulfisoxasole were lower in the post *vs*. pre regulation fall samples (odds ratio and *p*-value are presented in Table 3).

**Figure 7:**
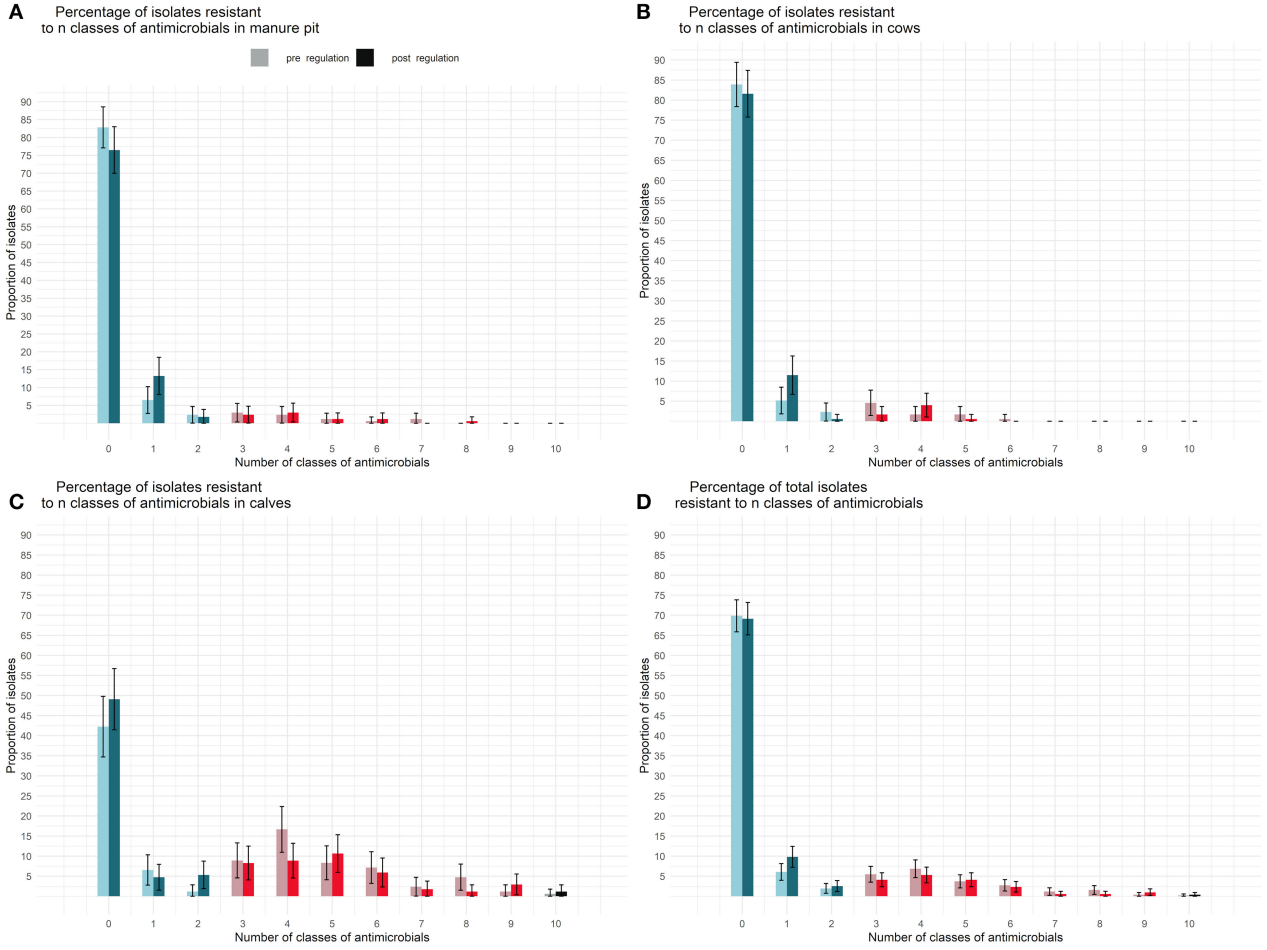
Comparison of the percentage of *E. coli* isolates resistant to n classes of antimicrobial per sample type [manure pit **(A)**, cows **(B)**, calves **(C)**, herd **(D)**] from 87 dairy farms from Québec, Canada, pre (light) and post (dark) regulation implementation. On each farm, between 4 and 6 *E. coli* were tested for each antimicrobial. In dark and light blue, isolates are non MDR. In dark and light red isolates are MDR. In gray and black isolates are extensively resistant.

The repartition of isolates resistant to a given number of classes of antimicrobials is presented in Figure 7. An isolate originating from a calf sample was, on average, resistant to 2.5 (CI95% 1.9–3.4) classes of antimicrobials before and 2.2 (CI95% 1.6–3.0) classes of antimicrobials after the regulation. There was no statistical difference between sample origin or between pre and post regulation periods in terms of number of resistances per isolate. Moreover, the genetic resistance profiles did not seem to have changed between the pre and post regulation periods.

#### ESBL/AmpC Collection

As illustrated in Figure 5, there was no statistical difference between the pre and post regulation period for the samples originating from manure pit, cows, calves, or for the herd in general, or per season, for the presence of a putative ESBL/AmpC *E. coli* (Supplementary Figure S4).

**Figure 8:**
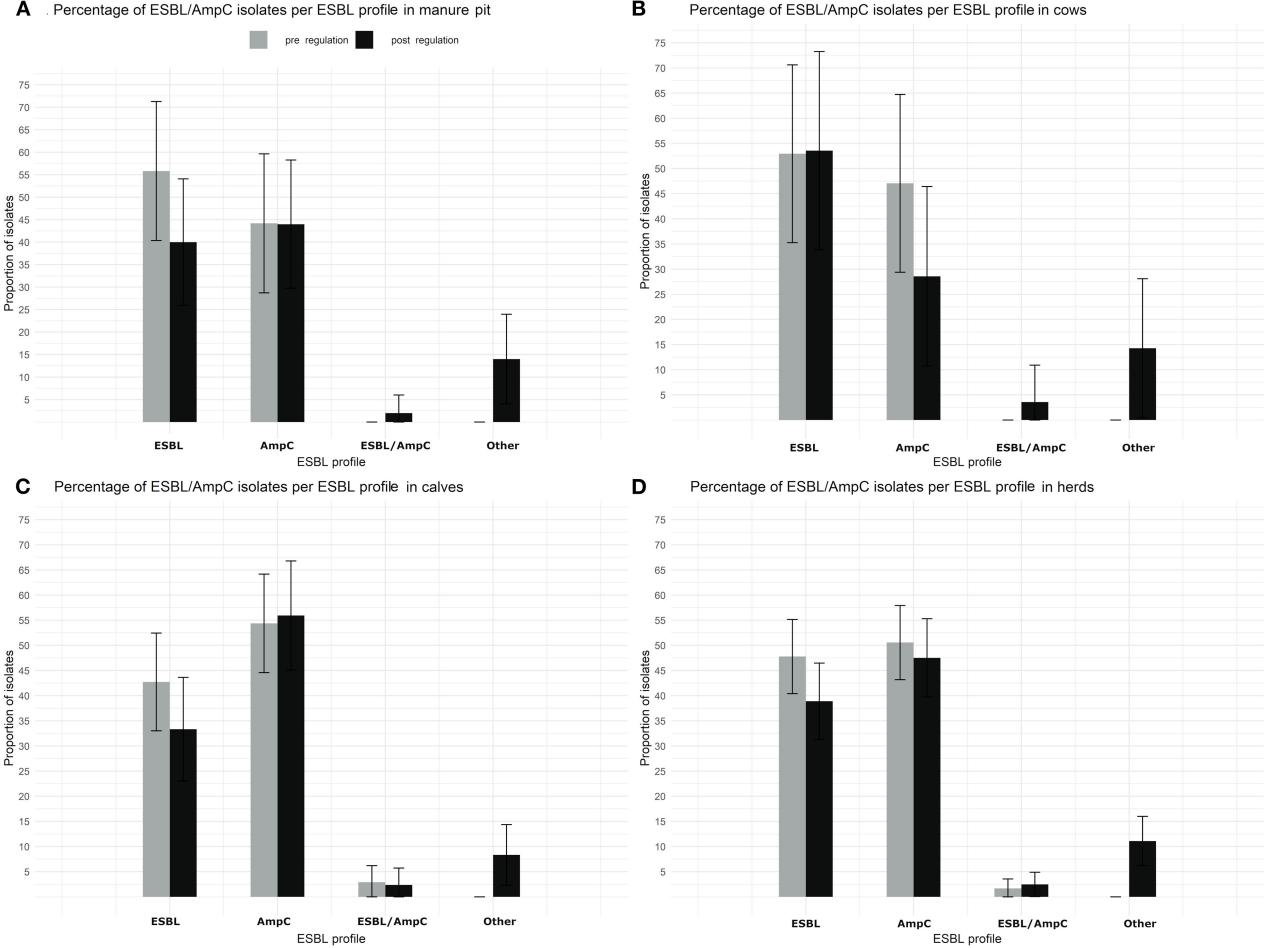
Comparison of the proportion of isolates with a putative ESBL/AmpC *Escherichia coli* per ESBL profile and per sample type (manure pit, cows, calves, herd) from 87 dairy farms from Québec, Canada, pre (gray) and post (black) regulation implementation. **(A)** Percentage of ESBL/AmpC isolates per ESBL profil in manure Pit. **(B)** Percentage of ESBL/AmpC isolates per ESBL profil in cows. **(C)** Percentage of ESBL/AmpC isolates per ESBL profil in calves. **(D)** Percentage of ESBL/AmpC isolates per ESBL profil in herds.

There was also no statistically significant difference in ESBL profile between pre and post regulation period, neither by sample type (Figure 8) nor by season (data not shown).

## Discussion

The main objective of this study was to establish the AMR situation in dairy cattle in Québec approximately 2 years after the implementation of a regulation limiting the use of category 1 antimicrobials according to Health Canada classification, and to compare this AMR situation to that of the period preceding the regulation implementation (9).

To the author's knowledge, Québec is a pioneer in Canada, regarding the implementation of a regulation restricting category 1 antimicrobial usage in production animals in February 2019. This study exploring the impact of such regulation on AMR in a Canadian context is also unique. Indeed, our research team was well-positioned to compare the AMR situation in dairy cattle in Québec post regulation implementation, as we collected data on AMR for a previous study in 2017, prior to the implementation of the regulation (9). The results of the present study demonstrate that the most significant decrease in the generic collection was for resistance to folate inhibitors and to aminoglycosides which led, consequently, to a decrease in MDR. On the other hand, we did not observe a significant decrease in resistance to any of the category 1 antimicrobials in the generic collection, nor in the ESBL/AmpC collection. These results are difficult to compare with any other previously conducted studies. Indeed, in 2016, several European countries, such as France and Belgium, had already banned the prophylactic use in animals of antimicrobials critical for public health, and the use of critical antimicrobial in production animal with some exceptions (such as emergency or if the veterinarian has proven with an antimicrobial susceptibility test that there is no other alternatives) (23, 24). In the Resapath annual report 2019 (25), which describes annually antimicrobial susceptibilities in animal pathogens from samples send to diagnostic laboratories in France, it seems that, in cattle, AMR toward cephalosporin and quinolones are decreasing. However, this tendency started in 2015 (prior to the ban), and statistical analyses were not performed on these data. Thus, it is difficult to attribute this impact to the regulation implementation vs. to the whole Ecoantibio plan (26). Indeed, this plan also involves the prevention of infectious diseases, the communication on AMR fight and the provision of tools to follow up on AMU. We found no studies assessing AMR post implementation in Belgium.

The lack of significant decrease in resistance to category 1 antimicrobials was to be expected for several reasons. First, the resistance to these antimicrobials was not very high in the first place. Second, category 1 antimicrobials are used mainly intramammary in bovine. Therefore, the impact of the regulation on the fecal microbiota might be low. Third, we recruited 87 farms to participate in the second study, therefore we might have been lacking power to detect a decrease in AMR. Fourth, although it is of great importance to have a thorough follow up of the situation, the time between the regulation implementation and the second period of sampling (~2 years) could be considered very short to capture a change in the AMR situation. There are very few data concerning the carriage duration for antimicrobial genes in cattle, as it is a complex question depending on the characteristics observed (genotype vs. phenotype), the variety of AMR, the mechanism of spread, the presence of co-selection, the selective pressure in the environment and even the microbiota of the individual animal (27). However, in humans the mean time to lose carriership of ESBL *E. coli* was determined to be 1.1 years (28). On the other hand, CTX-M ESBL-producing *E. coli* have been demonstrated to persist in fecal samples of calves for 69 weeks, specifically through the persistence of certain clonal lineages (29). A long-term AMR follow up is already planned, as a sentinel group of 30 dairy farms from Quebec (most of them were part of our study) was recently added to the Canadian Integrated Program for Antimicrobial Resistance Surveillance (CIPARS) program of the Public Health Agency of Canada (PHAC). Another explanation for the lack of significant decrease in resistance to category 1 antimicrobials may have been the context. Indeed, the COVID-19 pandemic started in February 2020 in Québec and has resulted in many supply difficulties, particularly for veterinary drugs. Consequently, several products were not available during this period, especially some products containing category 2 or 3 antimicrobials. Therefore, during certain periods, dairy farmers and veterinarians had no other alternative than to use a category 1 antimicrobial. On the other hand, a decrease of 80% of category 1 antimicrobial usage in the dairy industry in Québec was reported (10) during the same period. The impact of the regulation on category 1 usage could have been higher if not hampered by these logistic considerations.

The seasonal effect is interesting to note. Indeed, we found no significant decrease of AMR between the spring 2017 and the spring 2021. In general, the levels of AMR observed during the spring seasons were low, thus limiting the statistical power of the study. On the other hand, there was a significant decrease in resistance to folate inhibitors and aminoglycosides both at the herd level and for the calf samples between the fall of 2017 and the one of 2020. It is well-described that horizontal gene transfer, and therefore genome mobility, increases with the temperature (30, 31). It is possible that during the fall season (samples gathered during the early fall reflect what happened during the summer), resistance genes are more susceptible to antimicrobial pressure (even if there is no reported difference in the AMU between season). Indeed, as genome plasticity increases, the variation (gain or loss of genes) of AMR genes prevalence might as well-increases.

The decrease of AMR was greater in calf samples. Even if the mechanisms are not completely elucidated, it is well-recognized that calves are more at risk to shed AMR bacteria than adults (32). Therefore, statistically, the decrease (if any) was more likely to be significant in the calf group. Given that for cows and manure pits, distribution of resistances was low, the study power was limited for these specific samples. The sampling post regulation in spring was performed early in the year. Therefore, we were not able to sample the manure pits as they were still frozen, and we sampled the end of the drainpipe. It has been reported that fresh manure samples have a higher alpha and beta diversity than manure pits (33). However, the same study demonstrated that there were no significant differences in AMR genes abundance or diversity between fresh manure and the manure pit samples. These results are supported by the fact that we did not find any significant differences between manure pit samples at any time in our studies and confirms that our comparisons between periods and sample types are reliable.

The decrease in folate inhibitor and aminoglycoside resistance is somewhat surprising because the regulation did not concern these categories of antimicrobials. The folate inhibitor and aminoglycoside resistance genes are often found on plasmids (34, 35). Therefore, due to co-resistances (other resistance genes present on the same plasmids), modification of AMU can have indirect effect on AMR. Moreover, some genes can be responsible for resistance to several antimicrobial classes. Therefore, the restriction of a specific class of antimicrobial can have repercussions on resistance to other antimicrobial classes. Furthermore, the regulation in Québec (as in other European countries) was accompanied by several other measures that probably contributed to the decrease of AMR. In particular, the veterinarians had access to complete training on the judicious usage of antimicrobials by several members of our research team (JPR, SD, DF, MA) in 2018–2019. Field veterinarians that followed this training then supervised dairy producer training. Consequently, the entire dairy industry had access to complete information on better usage of antimicrobials. It is very likely that this training had a role in the decrease we observed between the two periods. The discontinuation of the sale of a very popular intramammary formulation containing dihydrostreptomycin at the end of 2020 might also explain the decrease in resistance to streptomycin. Indeed, it was the only available product containing streptomycin and labeled for use in the bovine in Québec. However, even if its use was very prevalent (8), it is unlikely to be the only reason for this decrease as it was applied via the intramammary route, thus targeting a relatively narrow compartment with a relatively light density of microorganisms.

The frequency of *E. coli* producing ESBL/AmpC was much higher in the ESBL/AmpC than in the indicator collection. This was to be expected as healthy animals shed ESBL/AmpC isolates in small quantities (36). This data demonstrates the importance of improving detection sensitivity using enrichment with cefotaxime to allow more accurate estimation of the proportion of positive farms. Based on our results, *bla*_*CTX*−*M*−55_ and *bla*_*CMY*−2_ seem to predominate and be linked, respectively to ESBL and AmpC phenotypes. Analysis of the fecal metagenome, to be able to quantify the genes present in the sample would be a good way to detect any decrease in resistance gene burden. The *bla*_*EC*_gene family was not detected in the previous study because they were not included in the Resfinder database. This family of genes are class C beta-lactamases and are found in *E. coli*. They are not well-documented and not often reported. However, they have been observed in various environments such as in samples collected from human and cattle in Alberta and associated with β-lactam resistance (37); and in Gambia in non-human primates (38) where the phenotype was not documented. In one of our isolates, the presence of *bla*_*EC*−14_ seems the best explanation for the resistance to ceftiofur. It is also noticeable that the *qnrS* family genes, responsible for plasmidic resistance to fluoroquinolones (39), were not detected in the generic collection in the previous study (9), but were detected in this study. It might be a random finding, but the *qnrS* family genes should be monitored further in the next years. Indeed, even though they are usually known to be associated with a low degree of resistance (39), in our study 3 isolates presented clinically significant resistance to enrofloxacin and ciprofloxacin with no known mutation of the *parC* or *gyrA* genes. The presence of the *qnrS* genes was the only fluoroquinolone resistance determinant we detected.

The investigation on AMR gene dissemination mechanisms is essential because it helps in the fight to tackle AMR. It is often hypothesized that the relief of antimicrobial pressure will result in the loss of AMR genes, as some experiments in the 1970's showed (40). However, the reality is far more complex. Indeed, some genes carried by plasmids may impose little pressure on host strains and might be preserved even if the antimicrobial pressure is relieved (41). Other plasmids might carry resistance to other antimicrobials or even to disinfectant or heavy metals which would allow the plasmid to stay in the bacteria even if the antimicrobial pressure is removed. Therefore, in case of plasmid carriage, other methods to diminish plasmid stability are needed to tackle persistence of AMR genes. According to our results, the dissemination mechanisms of resistance genes are a combination of clonal spread and horizontal gene transfer. First, three pairs of isolates (chosen among the most resistant in the generic collection) had the same phylogenetic characteristics (107412A and 106712A, 108511A and 102911A, 108112A and 106012A). The definition of a clone remains a challenge and depends on the method used to characterize the isolates (42). Based on cgMLST, which is a very discriminant method (21), they could be considered as clones. As they belong to different farms and periods, these data strongly suggest a clonal dissemination of the most resistant *E. coli* isolates in the dairy population in Québec. All these clones were identified in calf samples suggesting that calves are more susceptible to harbor MDR clones, as other studies have already proposed (29). The putative vectors for clonal dissemination are likely physical, such as transporters, material lending between farms, veterinarians, etc. The mechanisms by which calves harbor MDR clones are not clear but could be associated with greater levels of AMR such as microbiota immaturity and increased contact between individuals (32). It is very interesting to note that their replicon profiles and their resistance pattern (Figure 4 and Supplementary Figure S1) are not identical, indicating that, in addition to this clonal dissemination, plasmids are also spreading resistance genes. Many of the replicons identified in this study belong to plasmid families known to carry AMR genes, and to be epidemic plasmids (43). However, our method of sequencing did not allow us to study the plasmids in greater depth.

In conclusion, ~2 years after the regulation to limit the use of category 1 antimicrobials was implemented in the province of Québec, Canada in production animals, the proportion of MDR *E. coli* isolates decreased significantly in the dairy industry specifically due to a decrease in resistance to folate inhibitors and aminoglycosides. It is likely that the regulation and all other measures implemented to improve judicious use of antimicrobials played a role in this decrease. This study highlights the importance of monitoring the impact of such regulation to adjust restrictions and maximize their effectiveness. Also, the elucidation of AMR gene dissemination mechanisms is essential strengthen the fight to tackle AMR.

## Data Availability Statement

The whole genome sequences presented in this study can be found in online repositories. The names of the repository and accession number can be found at: https://www.ncbi.nlm.nih.gov/bioproject/ PRJNA783194 /.

## Ethics Statement

The animal study was reviewed and approved by Animal Use Ethics and the Research Ethics Committees of the Université de Montréal (20-Rech-2085). Written informed consent was obtained from the owners for the participation of their animals in this study.

## Author Contributions

MA, JF, DF, CA, SD, M-ÈP, JM, and J-PR conceived research project and designed experiments. ML, HL, and J-PR coordinated sample collection. ML conducted experiments. ML, SD, JF, and J-PR contributed to data analysis and interpretation. ML wrote the draft manuscript. All authors critically revised it and therefore contributed to the submitted version.

## Funding

This work was funded by a grant from the Agri-Food Innov'Action Program resulting from an agreement between the Ministère de l'Agriculture, des Pêcheries et de l'Alimentation du Québec and Agriculture and Agri-Food Canada (Project IA 119542 to J-PR, collaborators CA, SD, DF, HL, MA, JF, and M-ÈP. ML also received scholarships from the Université de Montréal.

## Conflict of Interest

The authors declare that the research was conducted in the absence of any commercial or financial relationships that could be construed as a potential conflict of interest.

## Publisher's Note

All claims expressed in this article are solely those of the authors and do not necessarily represent those of their affiliated organizations, or those of the publisher, the editors and the reviewers. Any product that may be evaluated in this article, or claim that may be made by its manufacturer, is not guaranteed or endorsed by the publisher.

## Supplementary Material

Supplementary Figure S1 Replicon profile identified by whole genome sequencing of isolates of the generic collection (*n* = 15) from calf or cow feces or manure pit of 87 dairy farms in Québec, Canada in 2020–2021.

Supplementary Figure S2 Comparison of the proportion of isolates with a least one resistant (intermediate and resistant combined) *Escherichia coli* per sample type [manure pit **(A)**, cows **(B)**, calves **(C)**, herd **(D)**] from 87 dairy farms from Québec, Canada, spring pre (light) and spring post (dark) regulation implementation. On each farm, between 4 and 6 *E. coli* were tested for each antimicrobial. In dark and light burgundy, MDR: multidrug resistant (resistant to 3 classes of antimicrobial or more). In dark and light red, critical high priority antimicrobials for human medicine. In dark and light blue, critical priority antimicrobials for human medicine. In dark and light yellow, high priority antimicrobials for human medicine. In dark and light green: antimicrobials important for human medicine, SPE: spectinomycin. The importance of antimicrobial for human medicine was defined according to World Health Organization.

Supplementary Figure S3 Comparison of the proportion of isolates with a least one resistant (intermediate and resistant combined) *Escherichia coli* per sample type [manure pit **(A)**, cows **(B)**, calves **(C)**, herd **(D)**] from 87 dairy farms from Québec, Canada, fall pre (light) and fall post (dark) regulation implementation. On each farm, between 4 and 6 *E. coli* were tested for each antimicrobial. In dark and light burgundy, MDR: multidrug resistant (resistant to 3 classes of antimicrobial or more). In dark and light red, critical high priority antimicrobials for human medicine. In dark and light blue, critical priority antimicrobials for human medicine. In dark and light yellow, high priority antimicrobials for human medicine. In dark and light green: antimicrobials important for human medicine, SPE: spectinomycin. The importance of antimicrobial for human medicine was mentioned according to World Health Organization.

Supplementary Figure S4 Comparison of the proportion of isolates with a putative ESBL/AmpC *Escherichia coli* per sample type (manure pit, cows, calves, herd) from 87 dairy farms from Québec, Canada, pre (gray) and post (black) regulation implementation. **(A)** Comparison between spring 1 (pre) and spring 2 (post) regulation. **(B)** Comparison between fall 1 (pre) and fall 2 (post) regulation. No significant differences were identified between groups.

Supplementary Table S1 Quality of assembly data of whole genome sequencing of isolates from the generic collection (*n* = 15) from calf or cow feces or manure pit of 87 dairy farms in Québec, Canada in 2020–2021.
