# Supplementary figures and images for: Impact of a Regulation Restricting Critical Antimicrobial Usage on Prevalence of Antimicrobial Resistance in Escherichia coli Isolates From Fecal and Manure Pit Samples on Dairy Farms in Québec, Canada
Source: Front Vet Sci. 2022 Feb 17;9:838498. doi: 10.3389/fvets.2022.838498 (PMC8893019; doi:10.3389/fvets.2022.838498)

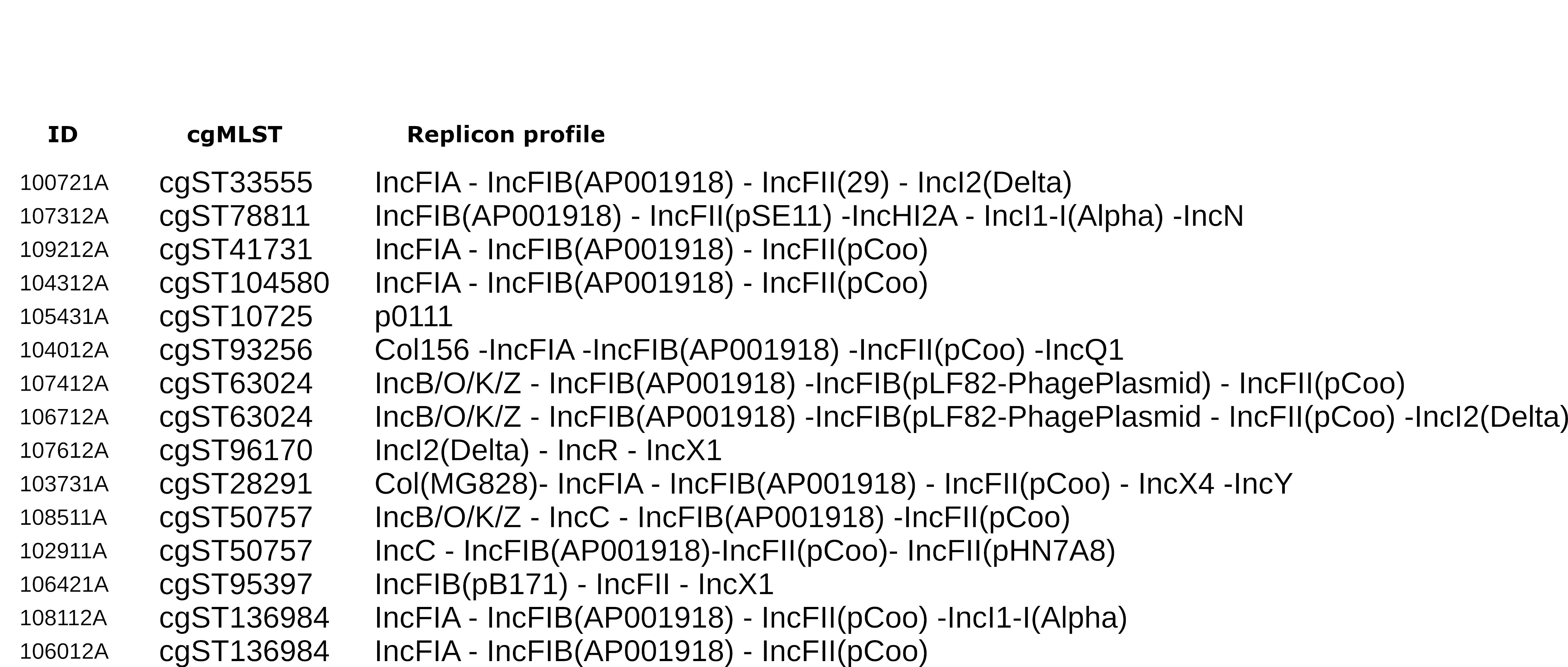

Supplement: Supplementary Figure S1 — Replicon profile identified by whole genome sequencing of isolates of the generic collection (n = 15) from calf or cow feces or manure pit of 87 dairy farms in Québec, Canada in 2020–2021. [file Image_1.JPEG]

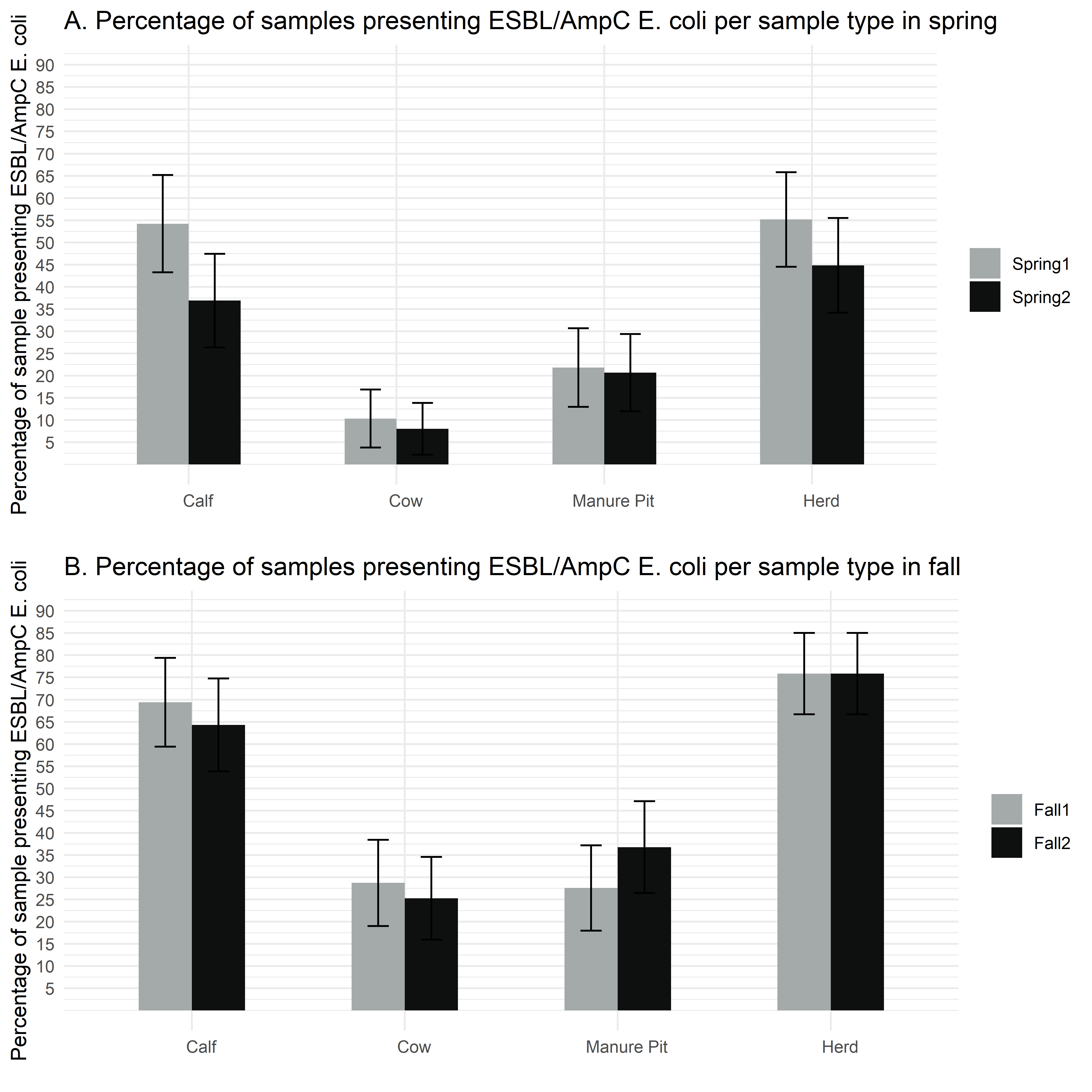

Supplement: Supplementary Figure S4 — Comparison of the proportion of isolates with a putative ESBL/AmpC Escherichia coli per sample type (manure pit, cows, calves, herd) from 87 dairy farms from Québec, Canada, pre (gray) and post (black) regulation implementation. (A) Comparison between spring 1 (pre) and spring 2 (post) regulation. (B) Comparison between fall 1 (pre) and fall 2 (post) regulation. No significant differences were identified between groups. [file Image_4.JPEG]
